# Very Low Serological Evidence of Exposure to *Francisella* spp. in Wild Boar and Red Deer from Central Portugal

**DOI:** 10.3390/pathogens15070675

**Published:** 2026-06-26

**Authors:** Humberto Pires, Sónia Saraiva, Manuela Matos, Ana Patrícia Lopes, Maria da Conceição Fontes, Cristina Pintado, Luís Figueira, Sérgio Santos-Silva, Ana Cristina Matos, Ana Cláudia Coelho, Luís Cardoso, João Rodrigo Mesquita

**Affiliations:** 1 Polytechnic Institute of Castelo Branco (IPCB), 6000-909 Castelo Branco, Portugal; humberto.s.pires@gmail.com (H.P.); cpintado@ipcb.pt (C.P.); lmftfigueira@gmail.com (L.F.); acmatos@ipcb.pt (A.C.M.); 2 Animal and Veterinary Research Centre (CECAV), University of Trás-os-Montes e Alto Douro (UTAD), 5000-801 Vila Real, Portugal; soniasaraiva@utad.pt (S.S.); aplopes@utad.pt (A.P.L.); mcfontes@utad.pt (M.d.C.F.); accoelho@utad.pt (A.C.C.); 3 Associate Laboratory for Animal and Veterinary Sciences (AL4AnimalS), 5000-801 Vila Real, Portugal; 4 Centre for the Research and Technology of Agro-Environmental and Biological Science (CITAB), University of Trás-os-Montes e Alto Douro (UTAD), 5000-801 Vila Real, Portugal; mmatos@utad.pt; 5 Centre Research for Natural Resources, Environment and Society (CERNAS-IPCB), 6001-909 Castelo Branco, Portugal; 6 ICBAS—School of Medicine and Biomedical Sciences, Porto University, 4050-313 Porto, Portugal; 7 Epidemiology Research Unit (EPIUnit), Instituto de Saúde Pública da Universidade do Porto, 4050-313 Porto, Portugal; sergiosilva.1999@hotmail.com (S.S.-S.); jmesquita@outlook.com (J.R.M.); 8 Laboratório para a Investigação Integrativa e Translacional em Saúde Populacional (ITR), 4050-313 Porto, Portugal

**Keywords:** *Francisella tularensis*, red deer, seroprevalence, surveillance, tularemia, wild boar, zoonosis

## Abstract

Tularemia is a zoonotic disease caused by the highly contagious bacterium *Francisella tularensis*, with rabbits, hares, and rodents considered as primary reservoirs. Clinical manifestations in humans can range from mild to life-threatening, depending on the mode of exposure (ingestion, inhalation, skin contact, injection, or tick bite), with high fever being a common feature. The primary aim of the present research was to evaluate the circulation of *F. tularensis* and the potential infection among wild boar (*Sus scrofa*) and red deer (*Cervus elaphus*) from the central region of Portugal. A total of 368 samples (including serum and organ samples) were collected from 184 wild boar and 184 red deer. For serological analysis, two commercially available enzyme-linked immunosorbent assay (ELISA) kits were used to detect immunoglobulin M (IgM) and G (IgG) antibodies to *F. tularensis*. Antibodies to *F. tularensis* (IgG and IgM) were detected in one adult male wild boar, corresponding to a seroprevalence of 0.54% (1/184, 95% confidence interval [95% CI]: 0.01–3.0%). No antibodies to *F. tularensis* were detected in red deer. Molecular detection by PCR was negative in the seropositive animal, in which submandibular lymph node, liver, and spleen samples were analysed targeting the 16S rRNA gene. These findings indicate exposure of wild boar to *F. tularensis* in central Portugal, suggesting a sporadic presence of the pathogen. Although no evidence of active infection was detected in the analysed tissues, the presence of seropositive individuals highlights the need for further investigation. Despite the very low seroprevalence observed, the zoonotic potential of *F. tularensis* supports the importance of continued surveillance within a One Health perspective.

## 1. Introduction

*Francisella tularensis* is a highly infectious, Gram-negative, facultative intracellular coccobacillus responsible for tularemia, a zoonotic disease of significant public health concern [1]. Tularemia is primarily a disease of the orders Lagomorpha and Rodentia, including hares, muskrats, rabbits, and squirrels [2]. The presence of *F. tularensis* has been detected in more than 250 animal species, including many other mammals and some birds [3], though the precise reservoir species are still not fully defined [4]. Small mammals, particularly lagomorphs and rodents, play a key role in pathogen amplification, often developing acute and fatal disease [5]. In contrast, other species, including wild ungulates such as wild boar (*Sus scrofa*) and cervids, are generally more resistant and may remain apparently healthy following infection, developing only a serological response [6]. Due to their feeding behaviour and wide ecological distribution, these species have been proposed as useful sentinels for monitoring environmental circulation of *F. tularensis* [7,8]. In Sweden, several wild predators and scavengers were serologically investigated, with seropositive individuals detected across multiple species, including wild boar (*S. scrofa*), suggesting that these animals may function as sentinels for tularemia [9]. In Portugal, wild boar may act as sentinel animals for zoonotic pathogens due to their role as a potential reservoir and increasing contact with humans and other animals [10]. Incursions of wild animals into urban areas amplify the potential risks of zoonotic disease transmission by increasing contact between humans and animal reservoirs [11]. In wild animals, the diagnosis of tularemia is most frequently established post-mortem during necropsy [2,8].

Four main subspecies of *F. tularensis*, each with distinct geographic distributions, have been identified in the world. *F. tularensis tularensis* (type A); *F. tularensis holarctica* (type B); *F. tularensis mediasiatica*, which occurs primarily in Central Asia and exhibits lower virulence; and *F. tularensis novicida*, a weakly virulent strain also present in the Northern Hemisphere but rarely associated with human disease. In general, infections in humans and animals are predominantly caused by *F. tularensis tularensis* (type A), mainly in Canada and the United States, and by *F. tularensis holarctica* (type B), which is distributed throughout the Northern Hemisphere, including Europe [1]. Human outbreaks are often sporadic and likely to be spatially and temporally variable, in addition to specific sites with potential for clusters of infection [12]. Transmission occurs through contact with infected animals (often hares), arthropod vectors (such as ticks or deer flies), inhalation of contaminated dust, or through contaminated food and water [13]. Human tularemia typically presents as an acute febrile illness with non-specific, influenza-like symptoms, including fever, lymphadenopathy, chills, fatigue, and headache, with clinical manifestations largely depending on the point of entry of the bacteria into the body [2,4].

In Portugal, *F. tularensis* subsp. *holarctica* has been detected in migratory shorebirds in 2012, suggesting that avian species may contribute to the dispersal of the bacterium or infected ticks across the peninsula [14]. This established presence in Portuguese territory is particularly relevant when considering the large outbreaks reported in northwestern Spain. In Castile and León region, the colonizing rodent *Microtus arvalis* exhibited a significant range expansion into agricultural landscapes between the 1970s and 1990s, playing a key role in tularemia epidemiology [15]. The irruptive population dynamics of this species act as an efficient amplification mechanism for the pathogen, being closely associated with human tularemia outbreaks [16].

The re-emergence of tularemia has been reported in recent years. Human cases have been reported mainly from the United States of America, Nordic countries like Sweden and Finland, and some other European and Asian countries [4]. Recent European reports describe increasing and geographically focal human tularemia, especially in Germany, Switzerland, Austria, Slovenia, and the Netherlands, with transmission commonly associated with arthropod bites [17,18], contact with hares or other wildlife (e.g., squirrel bites) [19], and, in some outbreaks, inhalation of contaminated dust [20] and consumption of infected hare carcasses [18]. In Austria, seroprevalence data revealed that tularemia infections are vastly underreported, with estimated incidence rates far exceeding notified cases, highlighting the need for improved surveillance and targeted prevention strategies [21]. In Germany, several particular features have been observed, including a broad genetic diversity of strains and the recent discovery of a new genus related to *F. tularensis*, as well as the occurrence of prolonged or more severe clinical courses associated with the subsp. *F. tularensis holarctica* [13]. The reemergence of this disease in Spain, after confirmation of *F. tularensis* in multiple wildlife species, along with epidemiological evidence of low antibody titres in sheep, dogs, and wild canids, suggests persistence of the pathogen in carrier hosts [22].

In 2023, 25 European Union (EU) member States reported a total of 1185 confirmed cases of tularemia, corresponding to a notification of 0.27 cases per 100,000 population. This represents an 89.3% increase compared with 2022, when the value was 0.14 per 100,000. In 2023, the highest notification values of tularemia in Europe were reported in Sweden (6.2 cases per 100,000 population), followed by Norway (2.7), Finland (1.9), and Switzerland (1.2). These findings highlight a marked geographical pattern, with the greatest burden observed in Northern Europe, particularly in Scandinavian countries, where tularemia remains endemic [23]. In Austria, the prevalence rose from 0.39 cases per 100,000 inhabitants to 0.58 in 2023, reaching 1.28 in 2024 [21].

Human tularemia in Portugal is rarely reported, with only a few documented cases, including both imported infections and a limited number of autochthonous cases, suggesting that the disease may be underdiagnosed [24,25].

### 1.1. Epidemiological Studies of F. tularensis in European Wild Boar and Deer

Epidemiological data on *F. tularensis* in European wildlife remain limited in European countries, particularly for wild ungulates such as wild boar and deer. Available studies report variable prevalence levels depending on geographic location and sampling strategies. The data compiled in Table 1 summarises reported occurrences of *F. tularensis* in wild boar and cervids across Europe, highlighting differences in detection values and study design.

Limited data are available regarding the occurrence of *F. tularensis* in wild boar and cervids across Europe. Serological investigations in wild boar indicate variable exposure, with prevalence ranging from 1.1% to 10.8%. However, molecular detection in carcasses generally reveals low or absent number of PCR-positive animals which may reflect either low levels of active infection or limitations related to sampling and detection methods [8,28]. A portion of the observed variation can be attributed to differences in geographic and temporal context, such that studies often capture distinct epidemiological foci rather than a homogeneous European baseline, while methodological factors further contribute to this variability (Table 1). When infections are spatially heterogeneous, sample sizes are limited, and detection depends on the sampled tissue or analytical method, focal infections may remain undetected. Consequently, serological surveys and carcass-based PCR assays, although related, address fundamentally different aspects of pathogen presence, which can result in markedly divergent prevalence estimates [29].

### 1.2. Surveillance of F. tularensis in European Wild Animals

Data reported in the EU One Health Zoonoses Report are derived from national surveillance systems submitted by EU Member States, integrating both human (ECDC) and animal (EFSA) data. In 2022, *F. tularensis* was detected by three EU Member States across five different animal categories. The majority of sampled animals in the EU (*n* = 363) were hares, of which 9.9% tested positive. Additional detections were reported in other animal groups, including monkeys, dogs, squirrels, moles, rabbits, and other rodents; however, these were based on very small sample sizes, with only a limited number of positive findings [30]. In 2023, *F. tularensis* was detected in three animal categories across three Member States. In the majority of cases, data were obtained through passive surveillance. Most of the samples tested in the EU originated from hares (*n* = 145), of which 20.7% were positive. Higher positivity rates were observed in other groups, including leporids (32.3%, *n* = 31) and dogs (50%, *n* = 8) [23]. Notably, passive surveillance of tularemia does not provide a comprehensive representation of the disease across entire animal populations. For instance, the positivity value reported in dogs in 2023 may not accurately reflect the true prevalence of the infection in this species, as the data are likely influenced by selective sampling and reporting biases [23]. The circulation of *F. tularensis* among wild animals has been reported in numerous countries across north-central Europe, where the pathogen is considered endemic [12,13,31], as well as in Spain [22].

In 2024, most of the animals tested were reported by Germany (92.4%; *n* = 2058), with 14.7% of animals testing positive, including 303 hares, one rabbit, and one wild boar. Austria recorded the highest positivity value at 30.8% (*n* = 16/52), followed by Sweden with 22% (*n* = 9/41) and Finland with 17.0% (*n* = 9/53). Switzerland reported data from pets, wild animals, and zoo animals, with 21.7% of samples testing positive (*n* = 5/23) [32].

Given the ecological complexity of tularemia and the potential role of wildlife as reservoirs (maintain and disseminate the pathogen in nature) or sentinels (indicate its presence through infection or immune response), seroepidemiological studies in wild ungulates can provide important insights into pathogen circulation and the associated risks for human exposure. In this context, the paucity of data on tularemia in wild ungulate populations represents a relevant knowledge gap, particularly considering the potential occupational exposure of hunters, game handlers, and veterinarians. From a One Health perspective, improved understanding of pathogen dynamics in wildlife is essential for assessing zoonotic risk and informing surveillance strategies. In Portugal, where sporadic human cases have been documented, information on the occurrence of *F. tularensis* in wildlife is limited, and the epidemiological significance of species such as wild boar and red deer remains unclear. The objective of the present study was to investigate the occurrence of *F. tularensis* in wild ungulates in Portugal using both molecular and serological approaches.

## 2. Materials and Methods

### 2.1. Study Area and Sample Collection

A cross-sectional study was conducted between 2016 and 2024 on free-range wild ungulates hunted during the official hunting seasons in central Portugal. The selected study areas included multiple municipalities representative of the primary distribution of wild ungulates in Portugal, located in the districts of Castelo Branco and Portalegre. The sampled municipalities comprised: Castelo Branco (*n* = 20), Castelo de Vide (*n* = 30), Crato (*n* = 28), Idanha-a-Nova (*n* = 59), Marvão (*n* = 22), Monforte (*n* = 7), Nisa (*n* = 31), Ponte de Sôr (*n* = 23), Portalegre (*n* = 9) and Vila Velha de Ródão (*n* = 139).

A total of 368 adult wild ungulates, representing the two species, comprised 184 wild boar (*S. scrofa*) and 184 red deer (*C. elaphus*). For each animal, whenever available, data regarding sex, age class, and clinical status were recorded.

Blood samples were obtained from the heart or thoracic cavity of the animals during the hunting season. Samples of tissue were collected from the spleen, liver, and lymph nodes from all animals. Blood was allowed to clot at environmental temperature, transported to the laboratory, and then centrifuged at 1500× *g* for 10 min. Serum samples were then stored at −20 °C until testing, which was performed shortly after collection.

### 2.2. Serological Analysis

Serological analysis was performed using two commercial enzyme-linked immunosorbent assay (ELISA) kits, SERION ELISA classic *Francisella tularensis* IgM and IgG (Institut Virion-Serion GmbH, Würzburg, Germany), to detect specific immunoglobulin M (IgM) and G (IgG) antibodies to *F. tularensis* in serum samples. According to the manufacturer, the assays show a sensitivity > 99% for both IgM and IgG, and a specificity of 96.4% for IgM and 99.0% for IgG in humans. For tularemia serology in wild boar and red deer, we followed previously described wildlife serology adaptation approaches, using a Protein G-HRP conjugate to broaden IgG recognition across mammalian species, consistent with published wildlife ELISA validations [33]. Optical density (OD) values were measured at 405 nm using an ELISA plate reader. Results were classified as positive, negative, or borderline according to the manufacturer’s instructions, based on index values (OD sample/calibrator). Samples were considered positive when the index exceeded 1.1 (≈OD > 0.57). Borderline samples were retested, and those remaining inconclusive were classified as negative.

### 2.3. Nucleic Acid Extraction

Total nucleic acids were extracted from 140 μL of each sample using the QIAamp DNA Mini Kit (Qiagen, Hilden, Germany), according to the manufacturer’s instructions. Extraction was performed on the automated QIAcube^®^ platform (Qiagen). The extracted DNA was eluted in nuclease-free water and stored at −80 °C until further analysis.

### 2.4. Molecular Detection of F. tularensis

PCR analysis was conducted exclusively on the seropositive animal and on a randomly selected subset of approximately 2% of animals from the parish of Rosmaninhal, using submandibular lymph node, liver, and spleen tissues. Individual samples were tested for molecular detection of *F. tularensis* using a conventional polymerase chain reaction (PCR) assay targeting the 16S rRNA gene and producing an amplicon of approximately 1450 bp. Amplification reactions were carried out using a T100™ thermal cycler (Bio-Rad, Hercules, CA, USA).

A positive control using *Francisella* DNA (GenBank Accession number MZ605214), previously detected by the group, was included in each PCR batch to verify assay performance. Negative controls consisting of PCR master mix with nuclease-free water (no-template control; NTC) were included in each PCR batch to detect potential contamination of reagents or reaction conditions.

PCR mixtures were prepared using the Speedy Supreme NZYTaq 2× Green Master Mix (NZYTech, Lisbon, Portugal), according to the manufacturer’s instructions, together with the primer pair previously described by [34], designed to detect *F. tularensis* as well as related *Francisella*-like bacteria:

Fr153F0.1: 5′-GCC CAT TTG AGG GGG ATA CC-3′

Fr1281R0.1: 5′-GGA CTA AGA GTA CCT TTT TGA GT-3′

The cycling conditions consisted of an initial denaturation at 95 °C for 5 min, followed by 40 cycles of denaturation at 94 °C for 2 s, annealing at 60 °C for 5 s, and extension at 72 °C for 5 s, with a final extension step at 72 °C for 10 min. PCR products were separated by electrophoresis on 1.5% agarose gels stained with Xpert Green Safe DNA gel dye (GRiSP^®^, Porto, Portugal). Electrophoresis was performed at 120 V for 30 min, and DNA bands were visualized under UV light.

### 2.5. Statistical Analysis

Seroprevalence was calculated as the proportion of positive animals among the total number tested, with corresponding 95% confidence intervals (CI). This calculation was done using two-sided exact binomial (95% CI) using GraphPad Prism 5.0 software (GraphPad Software, Inc., San Diego, CA, USA). Descriptive statistical analyses were performed to explore associations between *Francisella* spp. seropositivity and categorical variables (species, sex, age, and location).

## 3. Results and Discussion

A total of 368 wild ungulates were analysed by serology for the presence of *Francisella* spp., including wild boar (*n* = 184) and red deer (*n* = 184). The average number of animals tested per year ranged from 43 to 51 (mean ± SD: 46 ± 3.2), from 20 to 26 for wild boar (23 ± 2.0), and from 20 to 26 for red deer (23 ± 2.3), indicating low variation across the analyzed years. The serological analysis revealed evidence of IgG (0.74) and IgM (0.61) antibodies to *F. tularensis* in a single wild boar, corresponding to an overall seroprevalence of 0.27% (1/368; 95% CI: 0.01–1.5%).

The seropositive wild boar was identified in the municipality of Idanha-a-Nova (parish of Rosmaninhal). In this municipality, a total of 59 wild boar were tested, of which only one yielded a positive serological result in 2022. The seropositive animal was an adult male with no observable clinical signs of disease. PCR-based molecular analysis did not detect *Francisella* spp. DNA in the serologically positive animal or in any of the analysed samples.

The seroprevalence of antibodies (IgG and IgM) to *F. tularensis* in wild animals from Portugal is summarised in Table 2.

The seroprevalence of *F. tularensis* was very low in the analysed population, with only one positive animal out of 368 samples (0.27%), indicating rare exposure. A single positive case was detected in wild boar (0.54%), whereas no seropositive individuals were identified among red deer. Regarding sex, only one male was positive (1.41%), with no cases detected in females. Regarding age, the seropositive animal was an adult (0.60%), whereas no evidence of exposure was observed in juveniles. However, the very low number of positive cases limits the statistical power to detect associations with host factors such as sex and age. Overall, serological results indicated evidence of prior exposure within the studied population, whereas no *Francisella* DNA was detected by PCR in any of the analysed samples, including submandibular lymph nodes, liver, and spleen tissues.

These findings suggest limited or sporadic circulation of the agent, with no evidence of widespread transmission. However, given the detection of only a single seropositive animal and the absence of PCR-positive samples, the present study does not allow definitive conclusions regarding the epidemiological role of wild ungulates in tularemia dynamics in Portugal. While the findings are consistent with a limited role of these species, their potential involvement as incidental hosts (non-primary hosts that are infected accidentally and do not contribute to pathogen transmission), reservoirs or sentinels cannot be fully determined based on the available data. The low seroprevalence observed may reflect limited environmental circulation of the pathogen in the studied regions, potentially linked to low density of primary reservoir hosts, such as lagomorphs (0–50 *Oryctolagus cuniculus*/km^2^) [35], despite their presence in the region, or to ecological conditions that are unfavourable for pathogen persistence.

The discrepancy between molecular and serological results is consistent with previous reports on tularemia and reflects fundamental differences between diagnostic approaches [6]. Seropositivity indicates prior exposure and the development of a humoral immune response, which may persist for prolonged periods, whereas PCR detection is typically restricted to phases of active infection with sufficient bacterial load [6,36]. The probability of molecular detection may also be influenced by the selection of sampled tissues, as *F. tularensis* exhibits marked organ tropism, primarily localising in lymphoid tissues such as lymph nodes, spleen, and liver. Consequently, suboptimal tissue selection may reduce PCR sensitivity. This limitation is well recognised, as PCR performance depends strongly on the stage of infection, bacterial distribution, and the type of sample collected [37]. Additionally, antibody responses to *F. tularensis* typically develop in 2 to 3 weeks after infection and may persist for months or even years, whereas bacterial DNA is detectable only during a relatively narrow window of active infection, when sufficient bacterial loads are present in blood or tissues [31]. Therefore, animals sampled outside the acute phase of infection may yield negative PCR results, even in recommended tissue samples, due to low bacterial loads and the focal distribution of *F. tularensis*, despite evidence of prior exposure based on serology.

A limited number of studies have investigated the occurrence of *F. tularensis* in European wild boar and cervids, and the epidemiological role of these species remains poorly understood. Previous investigations have reported variable, but generally higher, seroprevalence in wild boar, ranging from 1.1% in central Germany [20] to 10.8% in the Czech Republic [7]. More recently, serological surveys conducted during the 2020–2023 hunting seasons in Denmark reported relatively high seroprevalence in cervids, particularly fallow deer (*D. dama*), reaching up to 40% [29]. These higher estimates likely reflect differences in geographic context, as these studies were conducted in endemic areas or regions with established tularemia circulation. In contrast, data from Portugal remain scarce, highlighting the relevance of the present study in contributing to regional knowledge. In the community of Castile and León, northwest Spain, from 2007 to 2020, outbreaks of tularemia were reported in humans, hares, wild rabbits, common voles and, to a lesser extent, field mice, shrews, and ticks. In addition, the low titres of antibodies to *F. tularensis* in sheep, dogs, and wild canids suggest the persistence of this pathogen in carrier hosts. However, the animal species or environmental niche acting as the true reservoir of *F. tularensis* could not be definitively identified [22]. Given the geographic proximity and ecological continuity between Spain and Portugal, particularly along shared border regions, these findings are of direct relevance to the Portuguese context. The documented epidemiological patterns in the Iberian Peninsula suggest that cross-border movement of wildlife, together with shared habitats and environmental conditions, may facilitate the introduction and/or circulation of *F. tularensis* between the two countries. Molecular studies have also identified positive cases in carcasses of wild boar and roe deer in France, where endemic tularemia transmission has been documented with hundreds of wildlife cases [8]. French surveillance revealed a marked disproportion between hare cases (693 confirmed) and ungulate cases (3 total), suggesting that European brown hares act as the primary reservoir, while infections in ungulates likely represent occasional spillover events. However, passive surveillance based on animals found dead may underestimate infection prevalence in ungulates, particularly because infections in these species are often mild or subclinical [8]. Tularemia cases in wildlife are spatially clustered and frequently associated with specific ecological niches, reinforcing the importance of environmental factors in pathogen maintenance. Additionally, the transmission dynamics of *F. tularensis* are closely associated with arthropod vectors, particularly ticks, whose distribution and abundance may strongly influence local exposure risk in wildlife populations [11,29]. Geographic expansion patterns have been documented, and tularemia may re-emerge after several years of apparent absence, likely driven by fluctuations in host populations, vector abundance, and environmental conditions. As *F. tularensis* potentially expands to new regions and host species [36], wild boar and deer populations in currently non-endemic areas may face increasing exposure risk.

Hunters are recognised as a risk group due to exposure through tick bites and carcass handling [36]. The reported human infections caused by the handling of a hunted hare indicate that this species has now become an established reservoir for *F. tularensis* in Western Austria [31]. However, reported human cases specifically linked to wild boar and deer remain limited. This may reflect the rarity of infection in these species, lower bacterial loads compared to primary reservoirs such as hares, or incomplete case attribution in public health surveillance systems. The absence of species-specific food safety guidance [28] suggests that the consumption of properly cooked wild boar and deer meat is not considered a major transmission route; however, robust evidence supporting this assumption remains limited. Nevertheless, the detection of seropositivity in wild boar highlights a potential zoonotic risk for individuals involved in hunting and carcass handling. In the present study, although prevalence is low (0.54% in wild boar), the high infectivity and potential severity of tularemia justify continued surveillance, even when only sporadic cases are reported.

One of the challenges in tularemia diagnosis is the limited sensitivity and specificity of many serological assays [37], and their performance under field conditions may be lower than that reported in controlled validation studies. The assays used in this study (SERION ELISA classic *F. tularensis* IgM and IgG) have a reported sensitivity > 99% for both IgM and IgG, and a specificity of 96.4% for IgM and 99.0% for IgG, according to the manufacturer. The positive serum sample identified in this study was retested, thereby reducing the likelihood of a false-positive result. However, false-negative results cannot be excluded, particularly in the early stages of infection or in cases with low antibody titres [37]. The limitations of the present study include the detection of only one seropositive animal, PCR testing performed on tissues for only a small number of animals and sampling restricted to hunted animals. Furthermore, there was an absence of environmental (e.g., water) and additional host sampling (e.g., ticks, lagomorphs and rodents), the inability to perform sequencing or genotyping and the lack of a confirmatory serological test (e.g., Western blot).

## 4. Conclusions

The detection of a seropositive animal, in the absence of PCR-confirmed case, may indicate possible exposure to *Francisella* spp. within the studied wild ungulate population, although this finding should be interpreted with caution given the low number of positive samples. These findings are consistent with previous European studies and underscore the importance of integrating serological and molecular approaches to accurately assess pathogen circulation. These results highlight the need for continued surveillance of *F. tularensis* in wildlife, particularly in regions currently considered non-endemic, to better understand its distribution, ecological drivers, and potential public health implications.

## Figures and Tables

**Table 1:** Epidemiology of *Francisella tularensis* in wild boar and cervids in Europe.

Year(s)	Country	Species	Method of Analyses	Total Animals Tested (Positive Results)	Study
1993–2001	Czech Republic	Wild boar	Serology (microagglutination)	204 (10.8% seroprevalence)	[7]
1995–2012	Central Germany	Wild boar	Serology (ELISA and WB)	566 (1.1% seroprevalence)	[26]
2005–2009	Germany	Wild boar	Serology (ELISA and WB)	80 (6% seroprevalence)	[27]
Hunting seasons (2002–2003 to 2012–2013)	France	Wild boar, roe deer	Carcasses (PCR on tissues)	3 PCR-positive animals (2 roe deer, 1 wild boar)	[8]
2011–2015	Sweden	Wild boar	Serology (slide agglutination and microagglutination)	248 (5.4% seroprevalence)	[9]
2015	Italy	Wildlife (red deer)	Carcasses (PCR on tissues)	60 (0 PCR-positive)	[28]
Hunting seasons (2020–2021; 2022–2023)	Denmark	Cervids (fallow deer, red deer, roe deer)	Serology (agglutination)	Fallow deer: 20 (40% seroprevalence); Red deer: 21 (23.8%); Roe deer: 253 (2.8%)	[29]

ELISA: enzyme-linked immunosorbent assay; PCR: polymerase chain reaction; WB: Western blot.

**Table 2:** Seroprevalence of antibodies to *Francisella tularensis* in wild animals from Portugal.

Animals	No. Anti-*F. tularensis* (Positive/Total)	% (95% CI)
Species		
Wild boar	1/184	0.54% (0.01–3.0%)
Red deer	0/184	0.00% (0.00–2.0%)
Sex		
Female	0/297	0.00% (0.00–1.2%)
Male	1/71	1.41% (0.04–7.6%)
Age		
Juvenile	0/201	0.00% (0.00–1.8%)
Adult	1/167	0.60% (0.02–3.3%)
Total	1/368	0.27% (0.01–1.5%)

CI: confidence interval.

## Data Availability

The original contributions presented in this study are included in the article. Further inquiries can be directed to the corresponding author.
